# Power and Efficiency Optimization for Open Combined Regenerative Brayton and Inverse Brayton Cycles with Regeneration before the Inverse Cycle

**DOI:** 10.3390/e22060677

**Published:** 2020-06-17

**Authors:** Lingen Chen, Huijun Feng, Yanlin Ge

**Affiliations:** 1 Institute of Thermal Science and Power Engineering, Wuhan Institute of Technology, Wuhan 430205, China; huijunfeng@139.com (H.F.); geyali9@hotmail.com (Y.G.); 2 School of Mechanical & Electrical Engineering, Wuhan Institute of Technology, Wuhan 430205, China

**Keywords:** combined cycle, inverse Brayton cycle, regenerative Brayton cycle, power output, thermal efficiency, finite time thermodynamics

## Abstract

A theoretical model of an open combined cycle is researched in this paper. In this combined cycle, an inverse Brayton cycle is introduced into regenerative Brayton cycle by resorting to finite-time thermodynamics. The constraints of flow pressure drop and plant size are taken into account. Thirteen kinds of flow resistances in the cycle are calculated. On the one hand, four isentropic efficiencies are used to evaluate the friction losses in the blades and vanes. On the other hand, nine kinds of flow resistances are caused by the cross-section variances of flowing channels, which exist at the entrance of top cycle compressor (TCC), the entrance and exit of regenerator, the entrance and exit of combustion chamber, the exit of top cycle turbine, the exit of bottom cycle turbine, the entrance of heat exchanger, as well as the entrance of bottom cycle compressor (BCC). To analyze the thermodynamic indexes of power output, efficiency along with other coefficients, the analytical formulae of these indexes related to thirteen kinds of pressure drop losses are yielded. The thermodynamic performances are optimized by varying the cycle parameters. The numerical results reveal that the power output presents a maximal value when the air flow rate and entrance pressure of BCC change. In addition, the power output gets its double maximal value when the pressure ratio of TCC further changes. In the premise of constant flow rate of working fuel and invariant power plant size, the thermodynamic indexes can be optimized further when the flow areas of the components change. The effect of regenerator on thermal efficiency is further analyzed in detail. It is reported that better thermal efficiency can be procured by introducing the regenerator into the combined cycle in contrast with the counterpart without the regenerator as the cycle parameters change in the critical ranges.

## 1. Introduction

A theoretical model of an open combined Brayton cycle (OCBC) was built by Chen et al. [1] on the bases of the models provided by Refs. [2,3,4,5,6,7,8,9,10,11,12,13,14,15]. In the OCBC model built in Ref. [1], an inverse Brayton cycle was introduced into regenerative Brayton cycle by resorting to the finite-time thermodynamics (FTT) [16,17,18,19,20,21,22,23,24,25,26,27,28,29,30], which has been applied for various processes and cycles [31,32,33,34,35,36,37,38,39,40]. The thermodynamic indexes of the OCBC have been analyzed in Ref. [1]. In order to further optimize the thermodynamic indexes, such as the power output (PO), thermal efficiency (TE), and pressure ratio (PR) of top cycle compressor (TCC), the analytical formulae related with 13 kinds of pressure drop losses (PDLs) are yielded. These PDLs take place in the whole cycle, such as the combustion chamber, the compressors, the regenerator, the turbines, as well as various flow processes. By employing the similar principle according to Refs. [41,42,43,44,45,46,47] and the method according to Refs. [2,3,4,5,6,7,12,13,14,15], the PO and TE will be numerically optimized in this paper.

In this paper, the performance optimizations of the OCBC will be conducted by means of varying the PR of TCC, mass flow rate (MFR), as well as PDL allocation. The maximum PO and TE of the OCBC will be gained after optimizations. Furthermore, the influences of cycle parameters on the optimal results will be numerically yielded.

## 2. Brief Introduction of the OCBC Model

**Figure 1:**
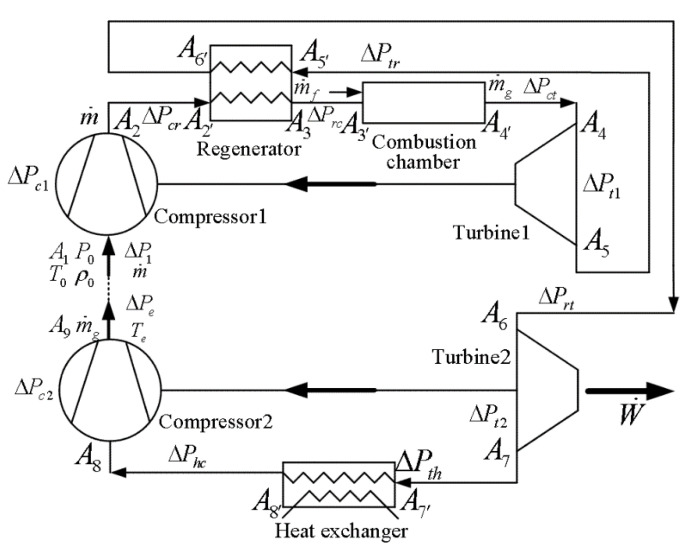
Pressure drop loss (PDL) and mass flow rate (MFR) distributions for the combined regenerative Brayton and inverse Brayton cycles [1,11].

Alabdoadaim et al. [11] proposed new configuration of an OCBC. It has a top cycle and a bottom cycle. The former is a regenerative Brayton cycle and is applied as a gas generator to power bottom one. The latter is an inverse Brayton cycle. The PO of the OCBC is totally produced by bottom cycle. As shown in Figure 1 [1,11], the top cycle contains compressor 1 (top cycle compressor (TCC)), regenerator, combustion chamber, and turbine 1 (top cycle turbine), whereas the bottom cycle contains turbine 2 (bottom cycle turbine), heat exchanger, and compressor 2 (bottom cycle compressor (BCC)).

According to FTT theory for open cycles [2,3,4,5,6,7,12,13,14,15], there are 13 kinds of flow resistances in the OCBC, 4 of them are evaluated by isentropic efficiencies of turbines and compressors, which take into account the friction losses in the blades and vanes, and the other nine kinds of them are caused by the cross-section variances of flowing channels, which exist at the entrance of TCC, the entrance and exit of regenerator, the entrance and exit of combustion chamber, the exit of turbine 1, the exit of turbine 2, the entrance of heat exchanger, as well as the entrance of BCC.

**Figure 2:**
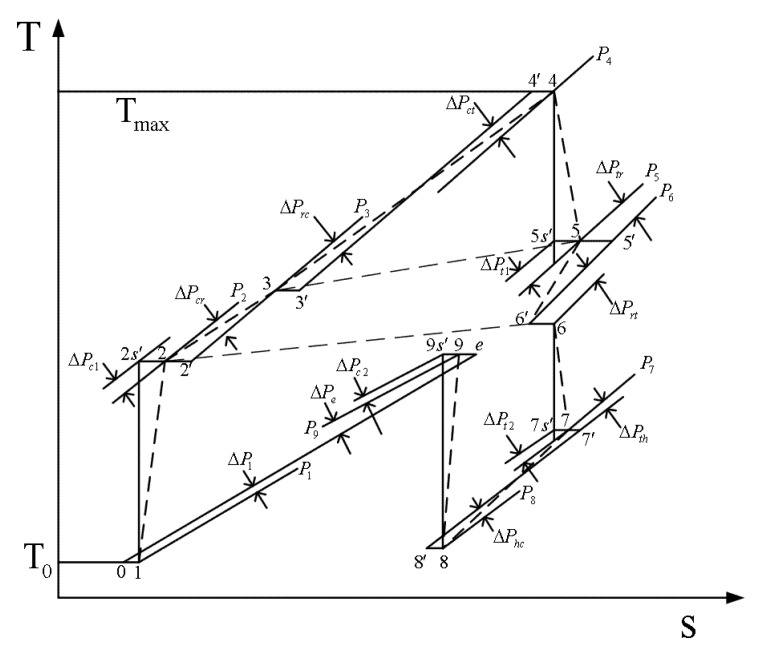
Temperature–entropy diagram and the flow resistances for the combined regenerative Brayton and inverse Brayton cycles [1].

The model of the OCBC, which is expressed using PDL and MFR distributions and temperature–entropy diagram, is shown in Figure 2 [1].

According to Chen et al. [1], after analyzing the OCBC, all of the PDLs in the system can be expressed as functions of the relative PD ($\psi_{1}$) of the entrance of TCC, $\psi_{1}=\Delta P_{1}/P_{0}$, where $P_{0}$ is the atmosphere pressure and $\Delta P_{1}=K_{1}(\rho_{0}V_{1}^{2}/2)$ is the PD of the entrance of TCC, where $K_{1}$ is the contraction pressure loss coefficient and $V_{1}$ is average air velocity through the entrance flow cross-section $A_{1}$ (see Figure 1).

Besides, all of the dimensionless power inputs of the compressors, power outputs of the turbines, as well as the heat transfer rate produced by fuel were obtained [1]; they are functions of the relative PD ($\psi_{1}$) of the entrance of TCC:

(1) $${\bar{W}}_{c1}=\frac{\gamma_{a1}\left(\theta_{c1s}-1\right)}{\eta_{c1}\left(\gamma_{a1}-1\right)}\psi_{1}{}^{1/2}$$

(2) $${\bar{W}}_{c2}=\left[1+1/\left(\lambda L_{0}\right)\right]\frac{\tau (T_{6}/T_{5})(\theta_{c2s}-1)\gamma_{gc2}}{\eta_{c2}(\gamma_{gc2}-1)\theta_{i}\theta_{t1}\theta_{t2}}\psi_{1}^{1/2}$$

(3) $${\bar{W}}_{t1}=\left[1+1/\left(\lambda L_{0}\right)\right]\frac{\eta_{t1}\tau (1-1/\theta_{t1s})\gamma_{g1}}{\gamma_{g1}-1}\psi_{1}^{1/2}$$

(4) $${\bar{W}}_{t2}=\left[1+1/\left(\lambda L_{0}\right)\right]\frac{\eta_{t2}\tau (1-1/\theta_{t2s})(T_{6}/T_{5})\gamma_{g2}}{(\gamma_{g2}-1)\theta_{t1}}\psi_{1}^{1/2}$$

(5) $${\bar{Q}}_{f}=\left(1+\frac{1}{\lambda L_{0}}\right)\frac{\gamma_{gc}(\tau -T_{3}/T_{0})}{(\gamma_{gc}-1)\eta_{cf}}\psi_{1}^{1/2}$$

where $\gamma_{a1}$ is air specific heat ratio, $\theta_{c1s}=T_{2s}/T_{1}=\beta_{c1}{}^{\left(\gamma_{a1}-1\right)/\gamma_{a1}}$ is isentropic temperature ratio of TCC, $\beta_{c1}=P_{2}/P_{1}=\beta_{1}/\left(1-\psi_{1}\right)$ is effective pressure ratio (PR) of TCC, $\beta_{1}=P_{2}/P_{0}$ is apparent compressor PR, $\eta_{c1}$ is isentropic efficiency of TCC; $L_{0}$ and λ are theoretical air quantity and excess air ratio of the combustor, $\tau =T_{4}/T_{0}$, $\gamma_{gc2}$ is gas specific heat ratio in turbine 2, $\theta_{c2s}=T_{9s}/T_{8}=\beta_{c2}^{\left(\gamma_{gc2}-1\right)/\gamma_{gc2}}$ is isentropic temperature ratio of turbine 2, $\beta_{c2}=P_{9}/P_{8}$, $\eta_{c2}$ is isentropic efficiency of BCC, $\theta_{i}=T_{7}/T_{8}$, $\theta_{t2}=T_{6^{\prime }}/T_{7}$, $\theta_{t1}=T_{4}/T_{5}$; $\eta_{t1}$ is isentropic efficiency of turbine 1, $\gamma_{g1}$ is gas specific heat ratio in turbine 1, $\theta_{t1s}=T_{4}/T_{5s}=\beta_{t1}{}^{\left(\gamma_{g1}-1\right)/\gamma_{g1}}$ is isentropic temperature ratio of turbine 1, $\beta_{t1}=P_{4}/P_{5}$; $\eta_{t2}$ is isentropic efficiency of turbine 2, $\gamma_{g2}$ is gas specific heat ratio in turbine 2, $\theta_{t2s}=T_{6^{\prime }}/T_{7s}=\beta_{t2}^{\left(\gamma_{g2}-1\right)/\gamma_{g2}}$ is isentropic temperature ratio of turbine 2, $\beta_{t2}=P_{6}/P_{7}$; $\gamma_{gc}$ is specific heat ratio in combustor; and $\eta_{cf}$ is combustor efficiency. All of the specific heat ratios for air and gas are evaluated according to empirical correlation based on averaged temperatures of air and gas [48,49].

According to two operation principle [11], one has ${\bar{W}}_{c1}={\bar{W}}_{t1}$. The net PO and TE are as follows [1]:

(6) $$\dot{W}={\dot{W}}_{t2}-{\dot{W}}_{c2}=\left[1+1/\left(\lambda L_{0}\right)\right]\{\frac{\eta_{t2}(1-1/\theta_{t2s})(T_{6}/T_{5})\gamma_{g2}}{(\gamma_{g2}-1)\theta_{t1}}-\frac{(T_{6}/T_{5})(\theta_{c2s}-1)\gamma_{gc2}}{\eta_{c2}(\gamma_{gc2}-1)\theta_{i}\theta_{t1}\theta_{t2}}\}\tau \psi_{1}^{1/2}$$

(7) $$\eta_{1}=\frac{(\gamma_{gc}-1)\eta_{cf}\tau }{\gamma_{gc}(\tau -T_{3}/T_{0})}\left[\frac{\eta_{t2}(1-1/\theta_{t2s})(T_{6}/T_{5})\gamma_{g2}}{(\gamma_{g2}-1)\theta_{t1}}-\frac{(T_{6}/T_{5})(\theta_{c2s}-1)\gamma_{gc2}}{\eta_{c2}(\gamma_{gc2}-1)\theta_{i}\theta_{t1}\theta_{t2}}\right]$$

## 3. Power Output Optimization

In this section, a series of numerical solutions are conducted to examine the influences of PR of bottom cycle, MFR of working air, as well as PDs on the net PO. In order to carry out numerical examples, the pertinent variation ranges and values of the cycle parameters are listed as: $0\leq \psi_{1}\leq 0.2$, $5\leq \beta_{1}\leq 40$, $1\leq \beta_{i}\leq 2.5$, 4≤τ≤6, $P_{0}=0.1013MPa$, $T_{0}=300K$, $\eta_{c1}=0.9$, $\eta_{c2}=0.87$, $\eta_{t1}=0.85$, $\eta_{t2}=0.83$, $\eta_{cf}=0.99$, ε=0.9, and $\varepsilon_{R}=0.9$ [2,3,11]. In addition, ratio of the outermost equivalent flow cross-sections (entrance of TCC/outlet of BCC) covered the range $0.25\leq a_{1-9}\leq 4$, where $a_{1-9}$ is the dimensionless group [1,2,3]:

(8) $$a_{1-9}=\frac{A_{1}}{A_{9}}{\left(\frac{K_{9}}{K_{1}}\right)}^{1/2}$$

(9) $$a_{1-i}=\frac{A_{1}}{A_{i}}{\left(\frac{K_{i}}{K_{1}}\right)}^{1/2},i=2,3,4,5,6,7,8,9$$

where $a_{1-2}=a_{1-3}=a_{1-5}=a_{1-6}=a_{1-7}=a_{1-8}=a_{1-9}=1/3$, $a_{1-4}=1/2$, and $0.25\leq a_{1-9}\leq 4$ are selected [1,2,3].

**Figure 3:**
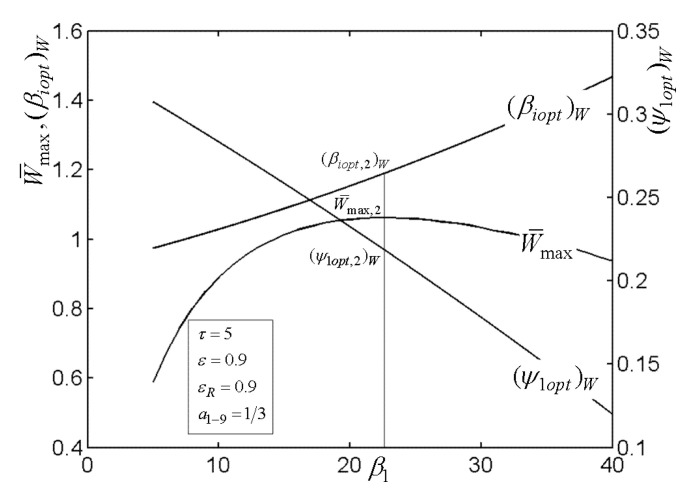
Relationships of ${\bar{W}}_{\max}-\beta_{1}$, ${\left(\beta_{iopt}\right)}_{W}-\beta_{1}$, and ${\left(\psi_{1opt}\right)}_{W}-\beta_{1}$.

**Figure 4:**
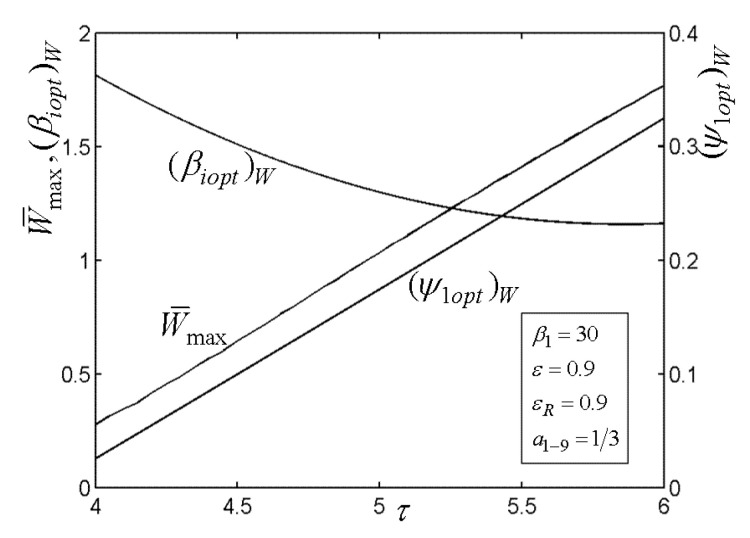
Relationships of ${\bar{W}}_{\max}-\tau$, ${\left(\beta_{iopt}\right)}_{W}-\tau$, and ${\left(\psi_{1opt}\right)}_{W}-\tau$.

**Figure 5:**
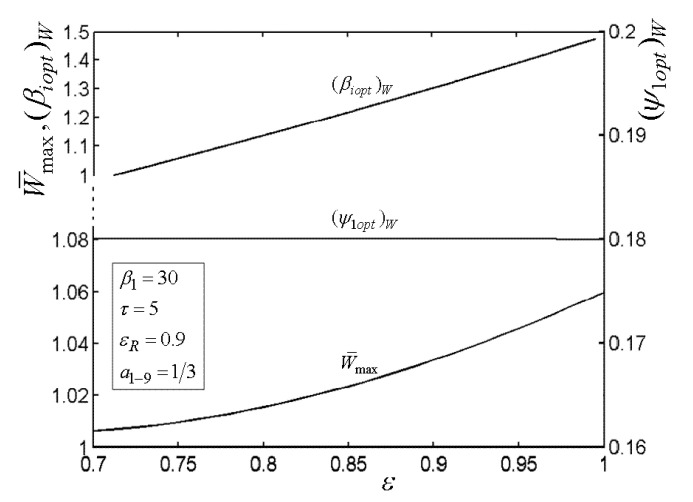
Relationships of ${\bar{W}}_{\max}-\varepsilon$, ${\left(\beta_{iopt}\right)}_{W}-\varepsilon$, and ${\left(\psi_{1opt}\right)}_{W}-\varepsilon$.

**Figure 6:**
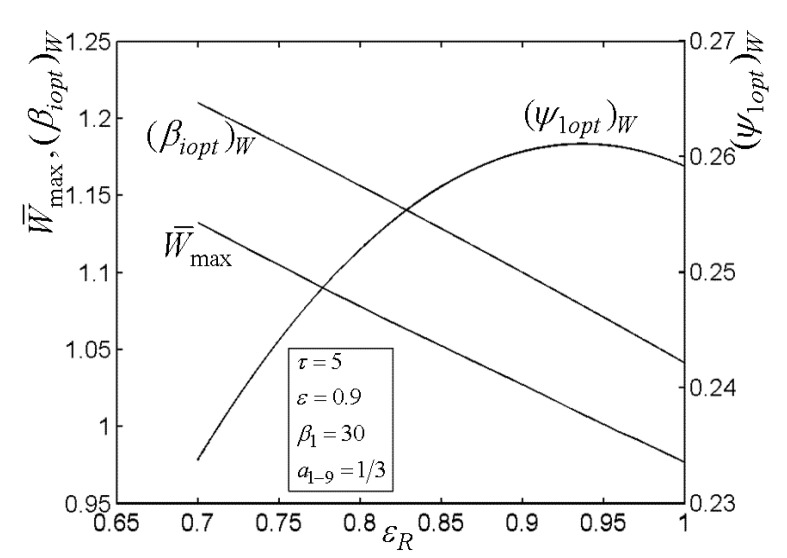
Relationships of ${\bar{W}}_{\max}-\varepsilon_{R}$, ${\left(\beta_{iopt}\right)}_{W}-\varepsilon_{R}$, and ${\left(\psi_{1opt}\right)}_{W}-\varepsilon_{R}$.

Figure 3, Figure 4, Figure 5 and Figure 6 present the relationships of the maximum dimensionless PO (${\bar{W}}_{\max}$) of the OCBC, relative optimal PR (${\left(\beta_{iopt}\right)}_{W}$) of BCC, as well as optimal entrance PD (${\left(\psi_{1opt}\right)}_{W}$) of TCC versus the PR ($\beta_{1}$) of TCC, temperature ratio (τ) of top cycle (TC), effectiveness (ε) of heat exchanger, as well as the effectiveness ($\varepsilon_{R}$) of regenerator, respectively. On the one hand, it is manifest that ${\bar{W}}_{\max}$ exhibits an increasing trend as τ and ε increase. However, it exhibits a decreasing trend as $\varepsilon_{R}$ increases. $\bar{W}$ can be twice maximized (${\bar{W}}_{\max,2}$) at the ${\left(\beta_{1opt}\right)}_{W}$. On the other hand, it can also be found that ${\left(\beta_{iopt}\right)}_{W}$ increases as $\beta_{1}$ and ε increase, while it decreases as τ and $\varepsilon_{R}$ increase. It is obvious that the relationships of ${\left(\psi_{1opt}\right)}_{W}$ versus $\beta_{1}$ and $\varepsilon_{R}$ exhibit the parabolic-like curves. ${\left(\psi_{1opt}\right)}_{W}$ increases as τ increases because the larger τ corresponds lager MFR of the working air.

**Figure 7:**
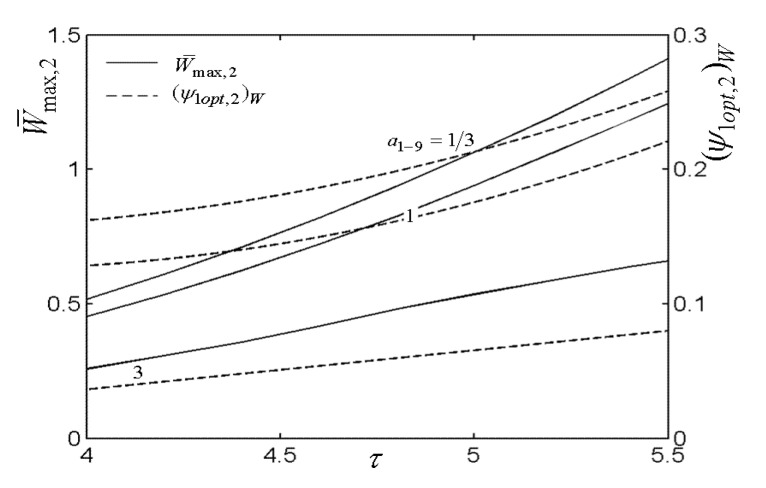
Influences of $a_{1-9}$ on the relationships of ${\bar{W}}_{\max,2}-\tau$ and ${\left(\psi_{1opt,2}\right)}_{W}-\tau$.

**Figure 8:**
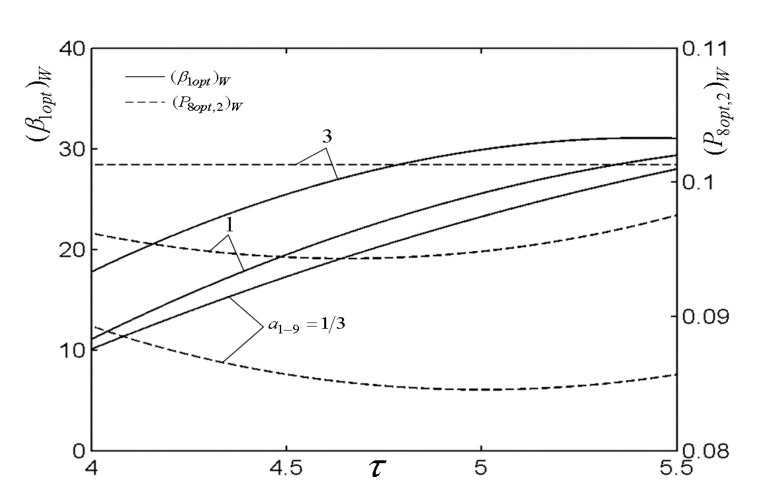
Influences of $a_{1-9}$ on the relationships of ${\left(\beta_{1opt}\right)}_{W}-\tau$ and ${\left(P_{8opt,2}\right)}_{W}-\tau$.

**Figure 9:**
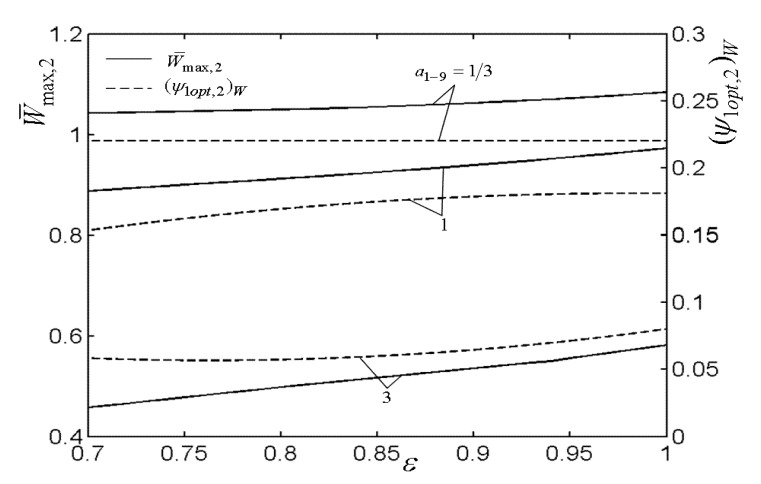
Influences of $a_{1-9}$ on the relationships of ${\bar{W}}_{\max,2}-\varepsilon$ and ${\left(\psi_{1opt,2}\right)}_{W}-\varepsilon$.

**Figure 10:**
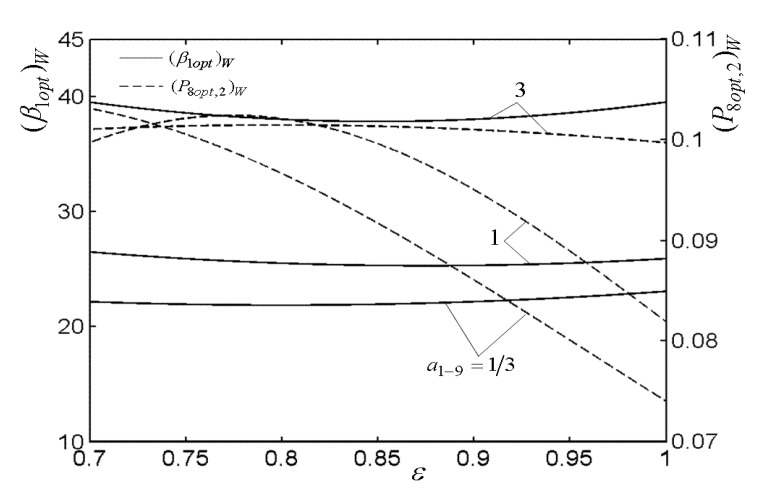
Influences of $a_{1-9}$ on the relationships of ${\left(\beta_{1opt}\right)}_{W}-\varepsilon$ and ${\left(P_{8opt,2}\right)}_{W}-\varepsilon$.

**Figure 11:**
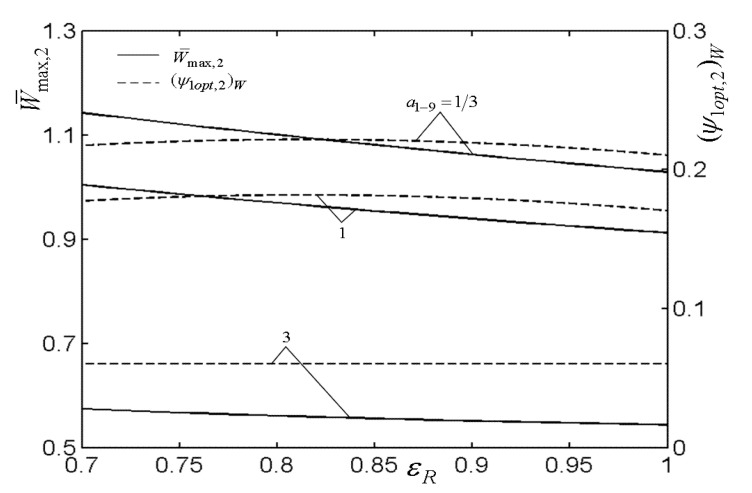
Influences of $a_{1-9}$ on the relationships of ${\bar{W}}_{\max,2}-\varepsilon_{R}$ and ${\left(\psi_{1opt,2}\right)}_{W}-\varepsilon_{R}$.

**Figure 12:**
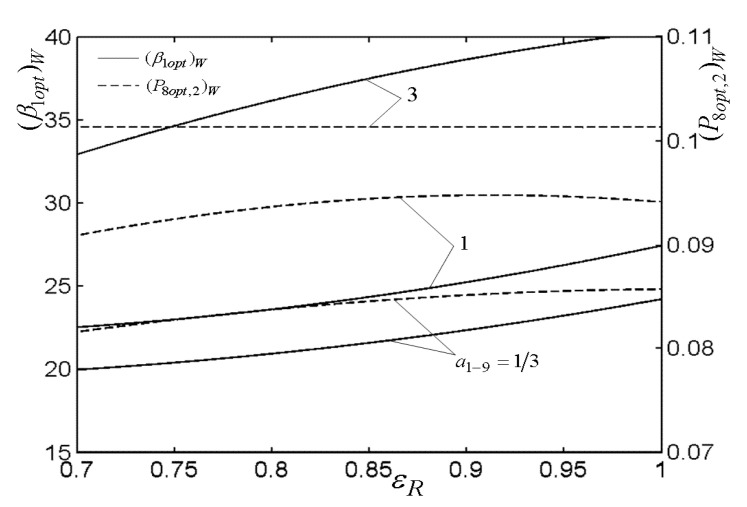
Influences of $a_{1-9}$ on the relationships of ${\left(\beta_{1opt}\right)}_{W}-\varepsilon_{R}$ and ${\left(P_{8opt,2}\right)}_{W}-\varepsilon_{R}$.

Figure 7, Figure 8, Figure 9, Figure 10, Figure 11 and Figure 12 present the influences of $a_{1-9}$ on the relationships of ${\bar{W}}_{\max,2}$, relative optimal PD (${\left(\psi_{1opt,2}\right)}_{W}$), ${\left(\beta_{1opt}\right)}_{W}$, as well as relative entrance pressure (${\left(P_{8opt,2}\right)}_{W}$) of BC versus τ of TC, effectiveness (ε) of heat exchanger, as well as effectiveness ($\varepsilon_{R}$) of regenerator, respectively. According to these figures for the fixed τ, ε, and $\varepsilon_{R}$, both ${\bar{W}}_{\max,2}$ and ${\left(\psi_{1opt,2}\right)}_{W}$ decrease as $a_{1-9}$ increases, and on the contrary, both ${\left(\beta_{1opt}\right)}_{W}$ and ${\left(P_{8opt,2}\right)}_{W}$ exhibit an increasing trend as $a_{1-9}$ increases. The twice maximized PO (${\bar{W}}_{\max,2}$) increases about 100% when $a_{1-9}$ decreases from 3 to 1 for the fixed τ. It shows that the size parameters of the entrance of TCC and outlet of BCC affect the performance of OCBC greatly. One can also see that ${\bar{W}}_{\max,2}$ exhibits an increasing trend as τ and ε increase, while it exhibits a decreasing trend as $\varepsilon_{R}$ increases. It shows that the regeneration cannot increase the PO in the discussed conditions because of the increase of PDL by adding a regenerator. In the case of $a_{1-9}=1/3$, ${\left(\psi_{1opt,2}\right)}_{W}$ increases as τ and ε increase. In addition, ${\left(\beta_{1opt}\right)}_{W}$ tends to gradually increase as τ and $\varepsilon_{R}$ increase. Besides, ${\left(P_{8opt,2}\right)}_{W}$ will be equal to environment pressure when $a_{1-9}$ is big enough. In this case, the BCC can be disregarded.

## 4. Thermal Efficiency Optimization

In this section, the aforementioned theoretical model is optimized herein by considering two practical constraints. The heat transfer rate (${\dot{Q}}_{f}$) discharged by working fuel is invariant. As a result, ${\dot{Q}}_{f}$ constraint is expressed by [1,2,3]

(10) $${\dot{Q}}_{f}=A_{1}{\left(2/K_{1}\right)}^{1/2}P_{0}{\left(RT_{0}\right)}^{1/2}Q_{f}\psi_{1}^{1/2}/(\lambda L_{0}RT_{0})=const$$

In addition, the other constraint is the total size of the OCBC, which is characterized by $A_{1}+A_{5}+A_{7}+A_{9}$. For simplification, the following constraint considering the areas ($A_{1}$ and $A_{7}$) of turbine 2 exit and TCC entrance is introduced [1,2,3]

(11) $$A_{1}/K_{1}^{1/2}+A_{7}/K_{7}^{1/2}=A_{*}=const$$

It is used to search for the optimal allocation ratio (x) of flow area defined by $A_{1}/K_{1}^{1/2}=xA_{*}$ and $A_{7}/K_{7}^{1/2}=(1-x)A_{*}$. From Equations (10) and (11), ${\bar{Q}}_{f*}$ is given as

(12) $${\bar{Q}}_{f*}={\dot{Q}}_{f}/\left[A_{*}P_{0}{\left(RT_{0}\right)}^{1/2}\right]=Cx\psi_{1}^{1/2}/\lambda =const$$

where $C=2^{1/2}Q_{f}/\left(L_{0}RT_{0}\right)$.

On this basis, the POs of turbine 2 and BCC can be, respectively, calculated as

(13) $${\bar{W}}_{t2*}=\frac{{\dot{W}}_{t2}}{A_{*}P_{0}{\left(RT_{0}\right)}^{1/2}}=\left[1+1/\left(\lambda L_{0}\right)\right]\frac{\sqrt{2}x\eta_{t2}\tau (1-1/\theta_{t2s})(T_{6}/T_{5})\gamma_{g2}}{(\gamma_{g2}-1)\theta_{t1}}\psi_{1}^{1/2}$$

(14) $${\bar{W}}_{c2*}=\frac{{\dot{W}}_{c2}}{A_{*}P_{0}{\left(RT_{0}\right)}^{1/2}}=\left[1+1/\left(\lambda L_{0}\right)\right]\frac{\sqrt{2}x\tau (T_{6}/T_{5})(\theta_{c2s}-1)\gamma_{gc2}}{\eta_{c2}(\gamma_{gc2}-1)\theta_{i}\theta_{t1}\theta_{t2}}\psi_{1}^{1/2}$$

From Equations (12)–(14), the TE derived by the first law of thermodynamics is written as

(15) $$\eta_{1}=\frac{{\bar{W}}_{t2*}-{\bar{W}}_{c2*}}{{\bar{Q}}_{f*}}=\frac{\sqrt{2}\lambda }{C}\left[1+\frac{1}{\lambda L_{0}}\right]\left[\frac{\eta_{t2}\tau (1-1/\theta_{t2s})(T_{6}/T_{5})\gamma_{g2}}{(\gamma_{g2}-1)\theta_{t1}}-\frac{\tau (T_{6}/T_{5})(\theta_{c2s}-1)\gamma_{gc2}}{\eta_{c2}(\gamma_{gc2}-1)\theta_{i}\theta_{t1}\theta_{t2}}\right]$$

**Figure 13:**
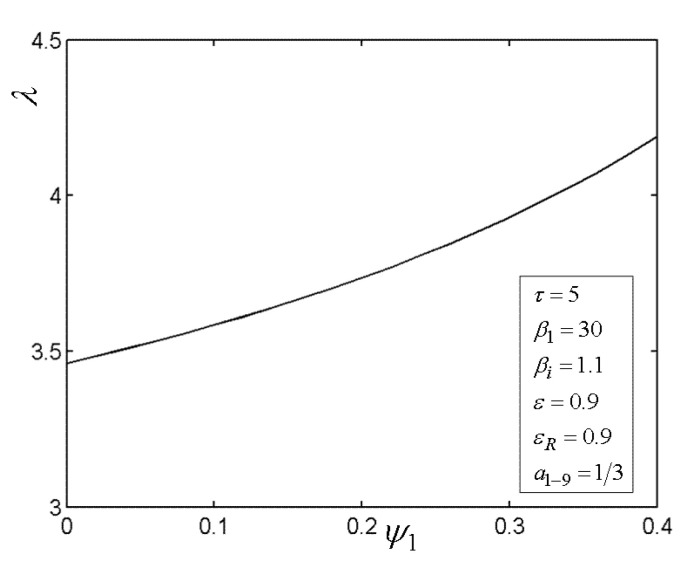
Relationships of $\lambda -\psi_{1}$.

**Figure 14:**
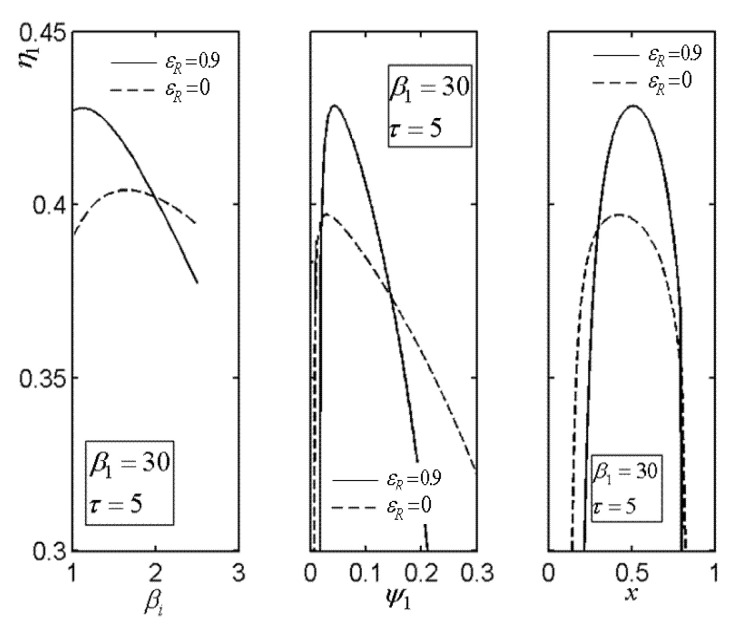
Influences of $\varepsilon_{R}$ on the relationships of $\eta_{1}-\beta_{i}$, $\eta_{1}-\psi_{1}$, and $\eta_{1}-x$.

Figure 13 presents the relationship of the excessive air ratio (λ) versus relative PD ($\psi_{1}$) of TCC entrance. As shown in Figure 13, it is indicated that λ increases as $\psi_{1}$ increases. Figure 14 presents the influences of regenerator effectiveness ($\varepsilon_{R}$) on the relationships of TE ($\eta_{1}$) versus PR ($\beta_{i}$) of BCC, relative PD ($\psi_{1}$) of TCC entrance, as well as area allocation ratio (x). As shown in Figure 14, it is indicated that $\eta_{1}$ can be maximized by selecting optimal values (${\left(\beta_{iopt}\right)}_{\eta }$, ${\left(\psi_{1opt}\right)}_{\eta }$ and $x_{opt}$) of $\beta_{i}$, $\psi_{1}$, and x in the both cases ($\varepsilon_{R}=0.9$ and $\varepsilon_{R}=0$). Moreover, in the discussed ranges of $\beta_{i}$, $\psi_{1}$, and x, the OCBC with regenerator can procure a better TE in contrast with the counterpart without regenerator. It shows that the regeneration can increase the TE.

**Figure 15:**
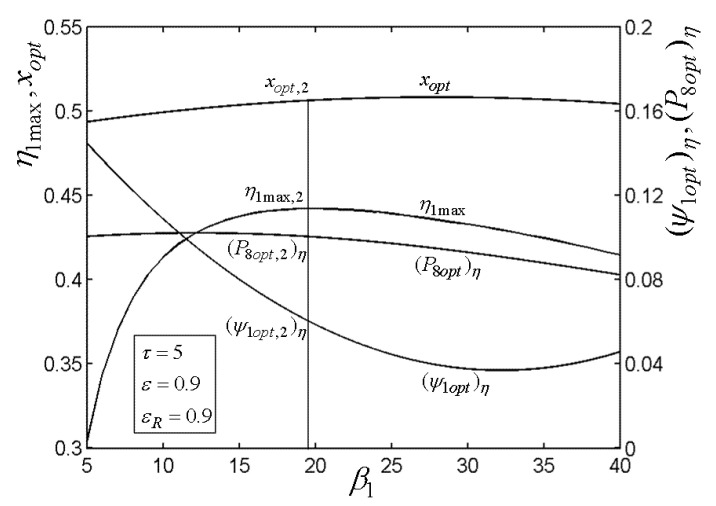
Relationships of $\eta_{1\max}-\beta_{1}$, $x_{opt}-\beta_{1}$, ${\left(\psi_{1opt}\right)}_{\eta }-\beta_{1}$, and ${\left(P_{8opt}\right)}_{\eta }-\beta_{1}$.

**Figure 16:**
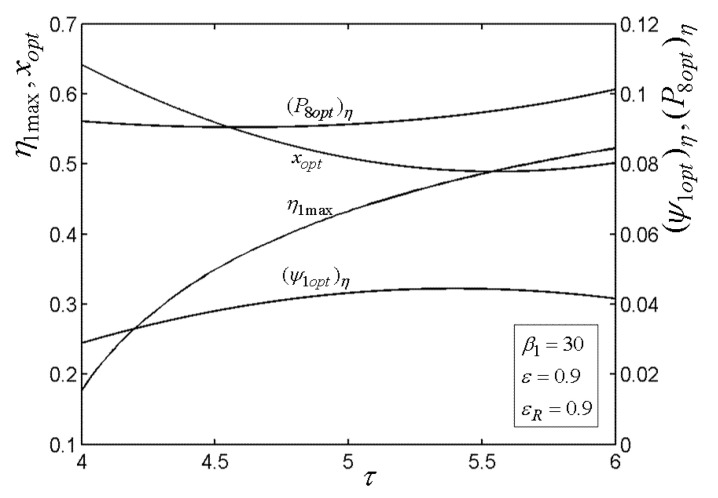
Relationships of $\eta_{1\max}-\tau$, $x_{opt}-\tau$, ${\left(\psi_{1opt}\right)}_{\eta }-\tau$, and ${\left(P_{8opt}\right)}_{\eta }-\tau$.

**Figure 17:**
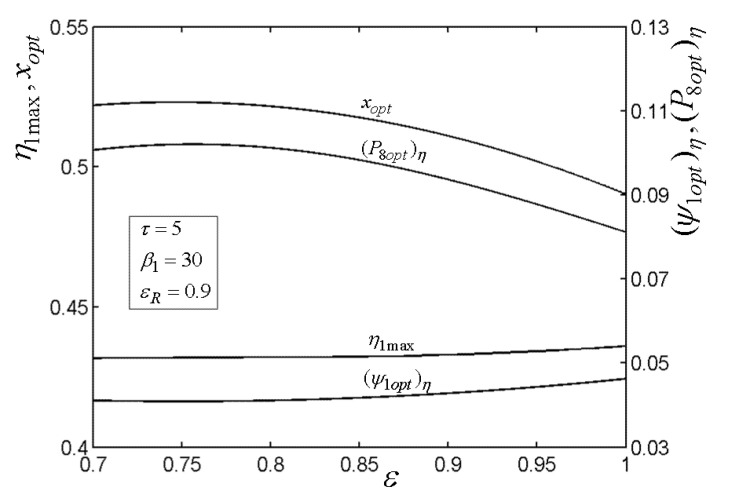
Relationships of $\eta_{1\max}-\varepsilon$, $x_{opt}-\varepsilon$, ${\left(\psi_{1opt}\right)}_{\eta }-\varepsilon$, and ${\left(P_{8opt}\right)}_{\eta }-\varepsilon$.

**Figure 18:**
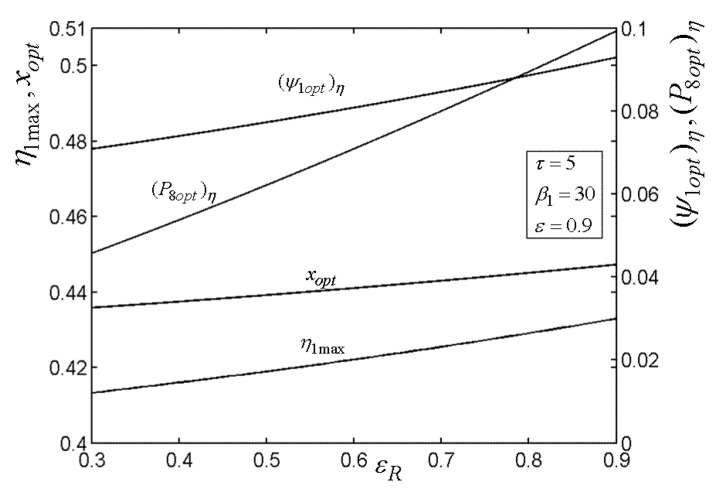
Relationships of $\eta_{1\max}-\varepsilon_{R}$, $x_{opt}-\varepsilon_{R}$, ${\left(\psi_{1opt}\right)}_{\eta }-\varepsilon_{R}$, and ${\left(P_{8opt}\right)}_{\eta }-\varepsilon_{R}$.

Figure 15, Figure 16, Figure 17, Figure 18 andFigure 19 present the relationships of the maximum TE ($\eta_{1\max}$), optimal PD ${\left(\psi_{1opt}\right)}_{\eta }$ of TCC entrance, optimal pressure ${\left(P_{8opt}\right)}_{\eta }$ of BCC entrance, as well as $x_{opt}$ versus the PR ($\beta_{1}$) of TCC, temperature ratio (τ) of TC, ε of heat exchanger, $\varepsilon_{R}$ of regenerator, as well as fuel constraint ${\bar{Q}}_{f*}$, respectively. According to these figures, it is manifest that $\eta_{1}$ can be twice maximized ($\eta_{1\max,2}$) at the optimal value ($\beta_{1opt}$) of $\beta_{1}$. Besides, $\eta_{1\max}$ exhibits an increasing trend as τ, ε, and $\varepsilon_{R}$ increase, while it exhibits a decreasing trend as ${\bar{Q}}_{f*}$ increases. One can also see that as $\beta_{1}$ increases, ${\left(\psi_{1opt}\right)}_{\eta }$ first decreases and then increases. However, ${\left(\psi_{1opt}\right)}_{\eta }$ always increases as τ, ε, $\varepsilon_{R}$, and ${\bar{Q}}_{f*}$ increase. It is shown that $x_{opt}$ exhibits an increasing trend as $\beta_{1}$ and ${\bar{Q}}_{f*}$ increase, while exhibits a decreasing trend as τ, ε, and $\varepsilon_{R}$ increase. In addition, one can also note that ${\left(P_{8opt}\right)}_{\eta }$ increases as τ, $\varepsilon_{R}$, and ${\bar{Q}}_{f*}$ increase, while it decreases as $\beta_{1}$ and ε increase.

## 5. Conclusions

In order to meet the increased request to the effective thermodynamic cycles, more and more new cycle models have been proposed in recently years. Agnew et al. [8] proposed combined Brayton and inverse Brayton cycles in 2003. Based on the combined Brayton and inverse Brayton cycles, Alabdoadaim et al. [9,10,11] proposed its developed configurations including regenerative cycle and reheat cycle and using two parallel inverse Brayton cycles as bottom cycles. The model cycle discussed in this paper was proposed by Alabdoadaim et al. [11] in 2006. They found that the regenerative combined cycle obtains higher thermal efficiency than that of the base combined cycle but smaller power output at small compressor inlet relative pressure drop of the top cycle based on the first law analysis. Chen et al. [1] established FTT model for this model cycle. This paper is to study the FTT performance in depth. Based on the OCBC model in Ref. [1], performance optimizations of the OCBC are conducted by means of varying the PR of TCC, MFR, as well as PDL allocation in this paper. The maximum PO and TE of the OCBC are gained after optimizations. Furthermore, the influences of cycle parameters on the optimal results are yielded. The numerical results reveal that:

1) Better TE can be procured by introducing the regenerator into the OCBC in contrast with the counterpart without the regenerator put forward by Ref. [7]. However, the performance of PO is inferior in the case of small PD of TCC entrance.
2) The net PO can be maximized by selecting the optimal PD of TCC and PR of BCC. Beyond this, the net PO can be twice maximized at the optimal PR of TCC.
3) The TE can be maximized by selecting the optimal PR of BCC. Additionally, it decreases as the PD of TCC entrance increases.
4) In the premise of constant rate of working fuel and total size of the power plant, TE can be maximized by selecting optimal values of $\beta_{i}$, $\psi_{1}$, and x. Furthermore, the TE can be twice maximized by varying the PR of TCC.
5) With consideration of area constraint of the flow cross-sections, TE can be maximized by reasonably selecting the flow areas of the components.
6) There exists optimal PD of TCC entrance. This means that there exist optimal MFR of the working air for the OCBC.

Although the discussed cycle model herein is not validated, the authors of this paper have studied other research objects and partially validated the theoretical models for open Brayton cycles [50,51]. Those can be seen as an illustration for the model herein.

## Figures and Tables

**Figure 19:**
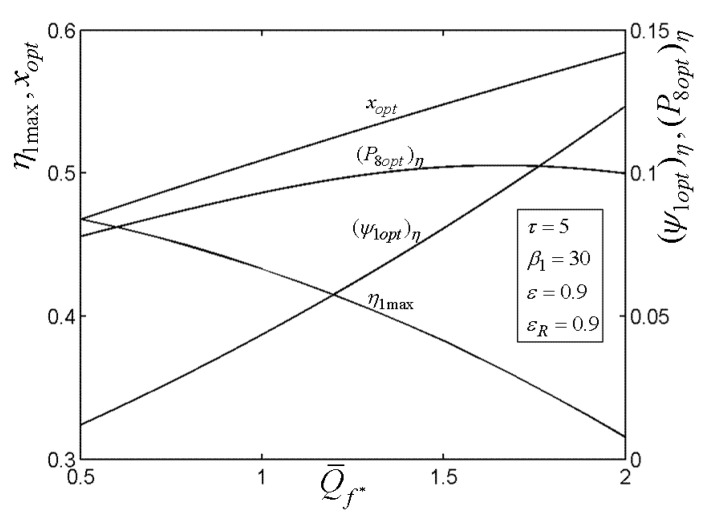
Relationships of $\eta_{1\max}-{\bar{Q}}_{f*}$, $x_{opt}-{\bar{Q}}_{f*}$, ${\left(\psi_{1opt}\right)}_{\eta }-{\bar{Q}}_{f*}$, and ${\left(P_{8opt}\right)}_{\eta }-{\bar{Q}}_{f*}$.

## Nomenclature

A	area
a	cross-section ratio
K	contraction pressure loss coefficient
L	excess air ratio
P	pressure
Q	heat
r	compression ratio
T	temperature
$\bar{W}$	power output
x	area allocation ratio
Greek symbol	
β	pressure ratio
ε	effectiveness
γ	ratio of specific heats
η	efficiency
λ	excessive air ratio
θ	adiabatic temperature ratio
τ	temperature ratio
ψ	pressure drop
Subscripts	
c	compressor
cf	combustor
f	working fuel
g	gas
max	maximum
opt	optimal
R	regenerator
t	turbine
0	ambient
1,2,3,…,9	state points in the cycle/sequence numbers
**Abbreviations**	
BCC	bottom cycle compressor
MFR	mass flow rate
OCBC	open combined Brayton cycle
PDL	pressure drop loss
PO	power output
PR	pressure ratio
TCC	top cycle compressor
TE	thermal efficiency
